# Erratum to “A single HIIT session does not alter blood sphingolipid levels in healthy young adults: The SphingoHIIT randomized controlled trial”

**DOI:** 10.34133/csbj.0071

**Published:** 2026-04-13

**Authors:** Thomas Angst, Nadia Weber, Seraina Fischer, Joëlle Lehmann, Denis Infanger, Tony Teav, Fabian Schwendinger, Lukas Streese, Timo Hinrichs, Ilaria Croci, Christian Schmied, Hector Gallart-Ayala, Christoph Höchsmann, Karsten Koehler, Henner Hanssen, Arno Schmidt-Trucksäss, Julijana Ivanisevic, Justin Carrard

**Affiliations:** ^1^ Division of Sport and Exercise Medicine, Department of Sport, Exercise and Health, University of Basel, Basel, Switzerland.; ^2^ Faculty of Biomedical Sciences, Universitá della Svizzera italiana, Lugano, Switzerland.; ^3^ Faculty of Medicine, University of Bern, Bern, Switzerland.; ^4^ Metabolomics Platform, Faculty of Biology and Medicine, University of Lausanne, Quartier UNIL-CHUV, Rue du Bugnon19, Lausanne, Switzerland.; ^5^ Faculty of Health Care, Niederrhein University of Applied Sciences, Krefeld, Germany.; ^6^ Department of Nephrology, Medical Faculty, Heinrich-Heine-University Düsseldorf, Düsseldorf, Germany.; ^7^ Cardiac Exercise Research Group, Department of Circulation and Medical Imaging, Norwegian University of Science and Technology, Trondheim, Norway.; ^8^ Sports Cardiology Section, Department of Cardiology, University Heart Center Zurich, University Hospital Zurich, University of Zurich, Zurich, Switzerland.; ^9^ Herzgefässzentrum im Park, Hirslanden Klinik im Park, Zurich, Switzerland.; ^10^ Department of Health and Sport Sciences, TUM School of Medicine and Health, Technical University of Munich, Munich, Germany.

In the Research article entitled “A single HIIT session does not alter blood sphingolipid levels in healthy young adults: The SphingoHIIT randomized controlled trial”, the authors identified an error in one of the supplemental files [1]. The raw data file (“SphingoHIIT_raw_data.xlsx”) included participant details that should have been removed. The unnecessary column has now been removed in the supplemental package in the original publication.

The authors apologize for the error.
